# Body weight, weight perceptions and food intake patterns. A cross-sectional study among male recruits in the Norwegian National Guard

**DOI:** 10.1186/1471-2458-11-343

**Published:** 2011-05-19

**Authors:** Solveig Uglem, Tonje H Stea, Wenche Frølich, Margareta Wandel

**Affiliations:** 1 Department of Nutrition, Institute for Basic Medical Sciences, University of Oslo, P.O Box 1046 Blindern, N-0316 Oslo, Norway; 2 Department of Public Health, Sport and Nutrition, Faculty of Health and Sport, University of Agder, 4604 Kristiansand, Norway; 3 Norwegian School of Hotel Management, University of Stavanger, N-4036 Stavanger, Norway

**Keywords:** weight perception, BMI, food intake patterns

## Abstract

**Background:**

Young men tend to have a low intake of vegetables and fruit. Unfortunately, this group is difficult to reach with health information. Furthermore, knowledge about weight perceptions and the relationship to food behaviour among young men is scant. The purpose of this study was to explore the relationship between BMI, health and weight perceptions and food intake patterns among young men in the military.

**Methods:**

Data were collected with a 4-day food diary among 578 male recruits (age 18-26, mean 19.7) in the Norwegian National Guard (response rate 78%), in addition to a questionnaire, including questions about health and weight perceptions, and food frequency when still living at home. Weight and height were objectively measured. Food patterns were explored with principal component analysis, based on the diary data. A multivariate linear regression analysis determined the association between BMI and food patterns, and attitudes to health and slenderness, adjusting for smoking, physical activity and phase of data collection.

**Results:**

Twenty eight percent of the recruits were overweight/obese (BMI > 25 kg/m^2^). Two-thirds meant that it is important for them to be slender, and these recruits reported more of both light (p = 0.025) and hard (p = 0.016) physical activity than the others. It was a positive association between the recruits' food frequency at home, and the amount of intake in the military camp for several food items. A principal component analysis identified three distinct food patterns, loading on 1) plant foods, 2) fast food/soft drinks, 3) milk/cereals. Those who stated that it is important for them to be slender, or to have good health, did not have significantly different food intake patterns than the others. BMI was inversely related to scores on the plant food pattern, and positive attitudes to slenderness.

**Conclusion:**

The majority of the recruits find it important to be slender. This orientation had a bearing on their physical activity pattern, but less on the food intake pattern. The data also indicate that subjects with high intakes of plant foods were less likely to have a high BMI than others. It is important to raise awareness of healthy eating in young men.

## Background

Young men are among the population groups that are most difficult to reach with health and nutrition information. They are also among the groups with the lowest intake of fruits and vegetables [1,2]. This is alarming since dietary patterns formed during early years may have implications for the risk of overweight/obesity and non-communicable diseases later in life [3,4]. Early adulthood, when moving away from home, is the time when changes in food behaviour are most likely to occur. However, there are also studies showing that dietary habits formed early in life track into adulthood [5-8].

There is limited knowledge about weight perceptions and how this is related to food behaviour among young men. Research has shown that men are less concerned about body image and weight than women [9,10]. In recent years, however, an increased concern about body image among men is observed. In a review from 2001, Cohane and Pope [10] observed that the ideal body image among boys varied; some boys had a thin ideal while others had a heavier one. Data from some Western societies indicate that a slender, but muscular body shape often is valued for men [11]. A survey among European university students showed restriction in intake of snacks among both men and women who were dieting [12]. However, studies have shown that women rely more on dieting and are more likely to avoid fattening food than men.

The aims of the present study were to assess food intake patterns, health and weight perceptions and BMI, and to explore the relationships between these aspects in a group of young men in the military service of Norway. The specific questions were: Does the food intake in the military reflect food consumption at home? How important is it for young men to be slender, and does the preference for slenderness influence the food and the physical activity patterns? To which extent do food patterns and attitudes towards health and slenderness relate to BMI?

A military camp is one of the best places to reach a broad variety of young men in Norway, as compulsory military service is practised (Act of 17 July 1953). At Vaernes Military Training Centre, where our data were collected, the recruits came from all over the country and all social classes were represented.

## Methods

### Participants

A total of 739 eligible recruits (aged 18 to 26) from The Norwegian National Guard at Vaernes, a military training centre near Trondheim, Norway, were asked to participate in the baseline surveys in January 2004 (n = 231) and July 2004 (n = 508). A total of 89 recruits refused to participate in the study. Of the remaining 650 subjects, 72 were excluded from the analysis due to incomplete data sets. The analyses are based on the remaining 578 subjects, 78% of the original sample.

### Setting

Most young men in Norway enrolled in the military service, come there directly after finishing high school and a life at home with their parents. Our data support this: 80% were 20 years or younger, and 94% had completed high school, but had no other education. Only 14 participants were above 23 years of age.

In most military camps in Norway, the soldiers have the opportunity to have free meals in a mess hall, or they can buy foods and beverages on their own expenses in a canteen. Both the mess hall and the canteen are located in the camp at Vaernes. The soldiers may also choose to buy foods, snacks and beverages in cafeterias/restaurants or grocery stores outside the military camp. Vending machines offering sugar-sweetened soft drinks and snacks were available at several places in the camp.

### Height, weight and body mass index

Body mass index, BMI, was computed from measured height and weight by personnel at Vaernes Military Training Centre, as weight/height^2 ^in kg/m^2 ^(Quetelet's index). BMI was classified into underweight (BMI < 18.5), normal (BMI 18.5 - 25), overweight (BMI 25-30) and obesity (BMI > 30), according to WHO [13].

### Survey instruments

Two survey instruments, a questionnaire about food habits, perceptions and attitudes and a diary, were used to assess the participants' consumption of foods and drinks and to identify their weight perceptions. The survey period started one week after enrollment (for both groups of enrollment) in the military service. The participants filled in the questionnaire on the first day in the survey period and completed the diary each day on four consecutive working days. Because it was important to keep the setting the same for all recruits, and the consumption at home and in the military separate from each other, they were asked to record food intake only on weekdays.

Both the diary and the questionnaire were tested in a pilot study with 12 soldiers in another military camp in Norway (Lutvann), and small amendments were made on the basis of the results and comments from this group. The participants did not report problems in remembering the frequency of consumption of the selected food items when still living at home. The revised survey instruments were test-retested with fairly acceptable responses over a three-week period among 63 recruits in a third military camp in Norway (Heistadmoen). Correlations between time 1 and 2 were from 0.11 to 0.55, however most responses were 0.50-0.55. Study procedures were approved from the necessary authorities; The Norwegian Social Science Data Services, The Ministry of Health and Care Services, and The Regional Committee for Medical Research Ethics.

### Consumption of foods and beverages

A validated diary developed at Department of Nutrition, University of Oslo http://www.helsedirektoratet.no/vp/multimedia/archive/00003/IS-1019_Ungkost_vedle_3757a.pdf was modified to assess the recruits' intake of selected food items like bread and other cereals, fruits, vegetables, meat and fish for lunch and dinner, in addition to fast food, snacks and beverages, as described in Uglem et al. [14]. The diary included pre-printed questions where the participants recorded their consumption during the day. The modification of the diary implied that the participants filled in a number for the chosen hot dish. Furthermore, to help participants determine serving sizes, the subjects were asked to refer to photographs or weighed portion-sizes placed on a table in the mess hall. Two researchers were also present at every meal to guide the recruits in determining serving sizes. The diary also included open-ended questions where the participants recorded their consumption of fast food, cakes/biscuits or snacks bought in the canteen in the camp or in groceries/cafeterias/restaurants outside the camp. These data gave the number of days on which the participants consumed fast food, cakes/biscuits or snacks in the survey period.

To estimate the ingredients included in hot dishes, recipes were collected from the kitchen personnel. Intake of beverages (soft drinks, juice and milk in g/day) was calculated based on the frequency intake of glasses of the beverage, multiplied by the serving size of a glass. One glass was defined as 150 gram. The intake of vegetables, fruits and cereals (in g/day) were calculated as the product of frequency of intake and serving sizes, and total consumption of bread as number of bread slices and sizes of the slices. Average daily consumption (g/day) of each food item for each recruit in the survey period was obtained by adding their total consumption and divide by the number of days on which each subject kept records. The recruits' consumption of fast food and snacks in the survey period was counted from the food diaries. The scale ranged from 0 to 4 days.

### Scales and coding

Weight perception was measured by the statement: 'It is important for me to be slender' and was scored on a five-point bipolar scale (strongly disagree to strongly agree). It was coded to two dichotomous variables; the first coded those who strongly agree versus the others. The second coded those who agree (strongly and to some extent) versus the others. Health perception was measured with the statement 'It is important for me to be in good health'. The response categories and coding were the same as for the question about slenderness. The time that the recruits presently spent in light or hard physical activity was measured with the question: "How would you describe your physical activity. Please give the average number of hours per week in each category of light and hard physical activity: Light activity (not sweating/out of breath); Hard activity (sweating/out of breath)". Their smoking habits were measured with questions about number of cigarettes/day and length of smoking, and recoded to current smokers and current non-smokers

Frequency of consumption of vegetables, fruit, breakfast cereals and snacks when still living at home, was collected through the question 'How often did you eat (the food) when you still lived at home'. Responses were measured on a six-point scale ranging from less than once a week to several times a day, and recoded to 3 categories 1) two or less servings/week 2) 3-6 servings/week 3) one or more daily servings.

### Data analysis

All analyses were performed using SPSS for windows, version 18. For description of participant characteristics and intake of different foods in the military camp, frequencies and means with 95% confidence intervals were reported, Pearsons χ2 test was used to analyse differences in consumption of snacks and fast foods in different groups, Independent samples t-tests were used to determine differences in means of food intake in grams, BMI in kg/m^2 ^and physical activity in hours/week between groups. ANCOVA was used to analyze differences between groups according to frequency of consumption at home and amount of consumption of the same foods in the military camp, adjusted for the phase of data collection.

A principal component analysis was carried out in order to study whether distinct food patterns could be discerned from the food intake data in the military camp. The factors were rotated by an orthogonal transformation. Both the eigenvalue (>1) and the scree plot was used to decide the number of factors extracted. In order to adjust for potential seasonal variation, the scores from each phase of data collection (January and July) were divided into tertiles which designated high, medium and low groups of scoring on the three food patterns. These were entered into a multivariate regression analysis in order to determine the association between BMI and food patterns, and attitudes to health and slenderness. The analysis was adjusted for possible confounders, such as smoking, physical activity. Since BMI may vary according to season, this model was also adjusted for phase of data collection.

## Results

### Participant characteristics

The recruits were between 18 and 26 years of age, with a mean age of 19.7 years (Table 1). The majority were within normal weight (BMI 18.5-25 kg/m^2^). Mean BMI was 23.2 kg/m^2^; 28.2% had BMI >25 kg/m^2^, including 4% that had BMI > 30 kg/m^2^. Thirty three per cent of the recruits were current smokers. About 40% reported that they had hard physical activity two hours per day or less, and 14% reported that they hardly performed any hard activity at all during the week.

**Table 1:** Participant characteristics (N = 578)

Variable	Recruits
Age, mean years, (CI)	19.7 (19.6-19.8)
BMI, mean kg/m^2^, (CI)	23.2 (22.9-23.6)
BMI <18.5, %	5.4
BMI 18.5-25, %	66.4
BMI >25-30, %	24.2
BMI >30, %	4.0
Smoking status, %	
Current smokers	33.2
Nonsmokers	66.4
Light physical activity, %	
<1 h/week	7.0
1-2 h/week	22.6
3-4 h/week	27.5
≥5 h/week	42.9
Hard physical activity, %	
<1 h/week	13.6
1-2 h/week	27.4
3-4 h/week	27.4
≥5 h/week	31.7

### Food consumption at home and in the military

The results showed a positive association between the recruits' frequency of consumption when still living at home and the amount of intake in the military camp with regard to several food items (table 2). Those who reported daily consumption of vegetables, fruits, breakfast cereals and sugared soft drinks when living at home, had considerable higher intake of these food items and drinks in the military camp, compared to those who had less consumption of these foods at home. Among those who had a daily consumption of snacks at home, there were 37% who bought snacks during the 4 days of registration, compared to 16% among those who had snacks less than once/week at home (not shown in the table).

**Table 2:** Frequency intake of selected food items at home related to amount intake in the camp, adjusted for phase of intake into the military.

Food intake at home Frequency of intake	Intake in the camp		p-value
	%	g/day	
**Vegetables**			
*< 1/week*	8	178	<0.001
*1 - 2 times/week*	22	229	
*3 - 4 times/week*	32	238	
*5 - 6 times/week*	18	254	
*Daily*	15	282	
*Several times a day*	6	284	
**Fruits**			
*< 1/week*	14	70	<0.001
*1 - 2 times/week*	31	95	
*3 - 4 times/week*	22	113	
*5 - 6 times/week*	14	168	
*Daily*	9	151	
*Several times a day*	10	225	
**Breakfast Cereals**			
*< 1 times/week*	65	55	<0.001
*1 - 2 times/week*	22	67	
*3 - 4 times/week*	6	152	
*5 - 6 times/week*	3	88	
*Daily*	2	178	
*Several times a day*	1	119	
**Semi- and whole grain bread**			
*Seldom*	5	125	<0.001
*1 - 2 slices/day*	10	143	
*3 - 4 slices/day*	28	156	
*5 - 6 slice/day*	29	170	
*7 - 8 slices/day*	15	192	
≤ *9 slices/day*	11	205	
**Soft drinks**			
*Seldom/never*	8	41	<0.001
*1 - 6 glasses/week*	40	74	
*1 glass/day*	18	107	
*2 - 3 glasses/day*	22	152	
≤ *4 glasses/day*	11	227	

### Attitudes towards health and slenderness

The majority of the recruits (70%) strongly agreed that it was important for them to have a good health. Furthermore, 31% strongly agreed, and 35% agreed to some extent that it was important for them to be slender. Thirty seven percent of those who strongly agreed that it was important for them to have good health, also strongly agreed that it is important for them to be slender. There were only small and insignificant differences in the consumption of snacks, fast food and soft drinks, between those who agreed (strongly or to some extent) that it was important for them to be slender, and the others. A higher intake of fruits was observed among subjects who agreed that it was important for them to be slender compared to those who disagreed (average difference 34 g/day (p < 0.05)). The same relationship was shown for juice, with a mean difference of 52 g/day (p < 0.05). Those who meant that it was important for them to be slender, reported more of both light (p = 0.025) and heavy (p = 0.016) physical activity than the others.

### Association between BMI and life style factors

Current smokers had a higher average BMI (24 kg/m^2^) than current non-smokers (never smoked and former smokers) (23 kg/m^2^), p < 0.05. There were no significant differences in BMI between those who reported high levels of physical activity and those who reported lower levels. The same was the case for hard or light physical activity.

Soldiers with BMI <25 kg/m^2 ^had a higher intake of bread (average difference of 25 g/day, p < 0.01) than those with BMI > 25 kg/m^2^. The same relationship was shown for vegetables (potatoes not included) (an average difference of 19 g/day, p < 0.05). For other food items no significant differences were observed.

### Food patterns, and the relationship to weight perceptions and BMI

Three main food patterns were observed among the recruits, based on their intake in the military camp (Table 3). A positive loading refers to a positive association between the food item and the factor, and a negative loading an inverse association [15]. The first pattern loaded positively on fruits, juices, potatoes, vegetables and bread, and was called *Plant foods*. The second pattern loaded positively on fast food and soft drinks and negatively on bread and was called *Fast food*. The last pattern loaded positively on all types of breakfast cereals and milk with an inverse association with snacks and was called *Milk/cereals*. Together these three patterns explained 42% of the total variance in the consumption of the selected food items among the recruits.

**Table 3:** Factor loadings for the three food patterns obtained in the study

Food item	Plant food Pattern 1	Fast food Pattern 2	Milk/cereals Pattern 3
Vegetables	0.70		
Potatoes	0.66		
Fruit	0.58		0.40
Juice	0.55		
Bread	0.35	-0.49	
Breakfast cereals			0.58
Milk			0.62
Fast food		0.70	
Candies	0.34		-0.55
Sugared soft drinks		0.75	

*Explained variance(%)*	17.76	13.84	10.64

The scores on the three food patterns obtained in the principal component analysis were divided into tertiles and used to make variables with the same names (Plant foods, Fast foods, Milk/cereals) which were then used in further analyses. No significant differences were found in the scores of these patterns between those who stated that it is important for them to be slender or to have good health, and the others.

A regression analysis with BMI as dependent variable showed a significantly negative relationship with the highest tertile of the plant food scale obtained in the factor analysis (Table 4). The results showed that subjects who had a higher score on factor 1 indicating higher intakes of vegetables, potatoes, bread, fruits and juices, were significantly more likely to have low BMI. The two other food patterns obtained in the factor analysis, showed no relationship with BMI. In addition, those who meant that it was important for them to be slender had a significantly lower BMI. The attitudes towards health were not significantly related to BMI.

**Table 4:** BMI in relation to food patterns and attitudes towards health and slenderness

	beta-value	p-value
Plant food (pattern 1)		
Medium tertile	-0.123	0.060
High Tertile	-0.171	0.012
Fast food (pattern 2)		
Medium tertile	0.055	0.401
High Tertile	0.093	0.154
Milk/cereals (pattern 3)		
Medium tertile	0.005	0.935
High Tertile	0.031	0.621
Attitudes towards health	0.055	0.348
Attitudes towards slenderness	-0.142	0.013
R	0.278	
R^2^	0.042	

Multivariate linear regression analysis. The 3 food patterns were entered as tertiles with the lowest tertile as reference. The 2 attitudes were entered as continous variables. The model was adjusted for smoking status (current smoker or not), light and hard physical activity (both in hours per week) and phase of data collection.

## Discussion

The data from the present study showed that slenderness was a highly valued attribute among the recruits; 66% agreed (strongly or to some extent) that it was important for them to be slender. Although the attitudes towards slenderness had more bearing on physical activity than on food behaviour, BMI was negatively associated with the intake of a plant food pattern defined using a principal component analysis.

The findings concerning the importance of slendernes are in line with studies in some other Western societies [11]. In the present study, the consideration for slenderness did not lead to notable restrictions in intake of fast food, snacks or soft drinks compared to those who disagreed with the statement. This is in agreement with the observation that young men are less likely to be on a diet than women of the same age [12]. This may explain why only small differences in intake of unhealthy foods were observed among the recruits with different perceptions about the importance of being slender. There is also a possibility for underreporting these types of food items, which may have affected this relationship.

The results from the present study showed that high consumers of unhealthy food items in the military camp also were high consumers of these foods when living at home. The same results were shown for healthy food items; those in the highest intake groups of fruits, vegetables and breakfast cereals at home continued to have higher intakes also in the camp. This suggests a substantial degree of stability in consumption habits even though they encountered a new environment. The data are in accordance with other studies from Norway reporting stability in food consumption from adolescence to young adulthood [5,7].

The principal component analysis identified three food patterns. These patterns had both similarities and differences compared with patterns derived in other studies [8,16]. The 'plant food' pattern had high positive factor loadings on food items considered healthy, like vegetables, potatoes, bread, fruits and juice. This pattern had some similarities with the 'prudent food pattern' derived in a study by Hu and co-workers [8] among men aged 40-75 years old. The mentioned factor loaded on vegetables, legumes, fruits, fish, poultry and whole-grain. The 'fast food' patterns in the present study, loaded on unhealthy food items such as fast food and soft drinks. This factor had similarities with the 'western diet pattern' observed in the study by Newby et al. [17], which had high loadings on red meat, refined grains, sweets, soft drinks and butter/margarine.

The average BMI in the present study was higher (+0.7 kg/m^2^) than in an earlier study of young men in the same age group in Norway [18], but lower than among American men in the same age group [19]. A relatively high proportion (28%) in the present study was classified as overweight or obese (BMI >25).

The regression analysis showed an inverse relationship between BMI and the the highest score on the plant food pattern. This indicates that subjects with a high BMI have a lower intake of fruits, vegetables, potatoes, bread and juices compared to subjects with low BMI. Those who reported that it is important for them to be slender, also had a lower BMI. Thus, it could be argued that the higher scoring on the plant food pattern among those with lower BMI could be due to restrained eating rather than the food pattern. However, the results indicated that those who agreed strongly that it is important for them to be slender, did not score higher on the plant food pattern. Other studies have shown similar results as found in the present study [16,17]. In the 1982-1997 FINRISK studies, daily consumption of vegetables were inversely associated with obesity (BMI > 30) among both men and women [16]. That study also showed that obesity was associated with smoking history.

A strength of our study is the way in which the dietary data were collected. The fact that the food analyses for the hot dishes were based on known recipes which included the amounts of all ingredients, that the glass sizes were known, and that recruits could use pictures and weighed portion sizes in the mess hall when reporting portion sizes, would most likely have increased the validity of the dietary data substantially. The guidance offered by the two research workers may also have increased the validity of the intake data. However, the food intake outside the mess hall would not be of the same high quality.

A limitation of the study is that the cross-sectional design does not provide evidence of a causal relationship between food consumption pattern and BMI. Another problem may relate to underestimation of food intake. Studies have shown that overweight and obese people tend to underestimate their food intake [20-22]. Nelson [22] has argued that subjects may choose not to record food items considered as unhealthy. This may have a bearing on the relationship between BMI and food intake patterns.

Furthermore, the fact that the data on food consumption in the military was measured on weekdays, whereas the data on food consumption at home referred to the entire week may have attenuated the association between these two measures of food consumption. However, this study showed a relationship between the intake at home and the intake in the military camp for many food items, suggesting some degree of continuity in food habits.

The use of BMI as an indicator of overweight or obesity has been questioned [15]. It is possible that some of the high BMI observed might be due to muscular mass rather than body fat, which could have blurred the relationship between BMI and physical activity. Another limitation of the study is the crude measurements of physical activity, which also could have blurred the relationship between BMI and physical activity.

In interpreting the results from the present study, some aspects of the setting and study sample should be noted. Firstly, the data were collected in a military camp where the subjects have some restrictions regarding their food and beverage choices. However, they have the possibility to have their meals both in the military mess hall and the canteen, and vending machines with soft drinks were available on several places in the camp. They could also buy foods, snacks and beverages outside the military camp. Secondly, limited generalisability of the results may be due to the fact that data were collected in just one military camp. However, compulsory military service is practised in Norway and young men from all over the country and all social classes are in the same military camp, and thereby represented in the study sample.

## Conclusions

The findings indicate that the majority of the recruits find it important to be slender. This orientation seems to have a bearing on their physical activity pattern, but less on the food intake pattern. Further, the study demonstrated that a high intake of some food items at home had a tendency to continue also in the military camp. In the principal component analysis a factor loading on plant foods was derived and further analysis showed that subjects with high intakes of plant foods had low BMI. More studies are needed to investigate the stability in food patterns over time, and to investigate the relationship between food patterns and risk factors for chronic diseases.

## Competing interests

The authors declare that they have no competing interests.

## Authors' contributions

WF and MW were responsible for the study concept and design. SU and THS acquired the data. SU was responsible for analysis and drafting of the manuscript. All authors participated in interpretation of data and critical revision of the manuscript for important intellectual content.

## Pre-publication history

The pre-publication history for this paper can be accessed here:

http://www.biomedcentral.com/1471-2458/11/343/prepub
